# Motor-Imagery EEG-Based BCIs in Wheelchair Movement and Control: A Systematic Literature Review

**DOI:** 10.3390/s21186285

**Published:** 2021-09-19

**Authors:** Arrigo Palumbo, Vera Gramigna, Barbara Calabrese, Nicola Ielpo

**Affiliations:** 1 Department of Medical and Surgical Sciences, “Magna Græcia” University, 88100 Catanzaro, Italy; palumbo@unicz.it (A.P.); calabreseb@unicz.it (B.C.); ielpon@unicz.it (N.I.); 2 Neuroscience Research Center, Magna Græcia University, 88100 Catanzaro, Italy

**Keywords:** motor-imagery (MI), brain-computer interface (BCI), electroencephalography (EEG), brain controller wheelchair (BCW)

## Abstract

The pandemic emergency of the coronavirus disease 2019 (COVID-19) shed light on the need for innovative aids, devices, and assistive technologies to enable people with severe disabilities to live their daily lives. EEG-based Brain-Computer Interfaces (BCIs) can lead individuals with significant health challenges to improve their independence, facilitate participation in activities, thus enhancing overall well-being and preventing impairments. This systematic review provides state-of-the-art applications of EEG-based BCIs, particularly those using motor-imagery (MI) data, to wheelchair control and movement. It presents a thorough examination of the different studies conducted since 2010, focusing on the algorithm analysis, features extraction, features selection, and classification techniques used as well as on wheelchair components and performance evaluation. The results provided in this paper could highlight the limitations of current biomedical instrumentations applied to people with severe disabilities and bring focus to innovative research topics.

## 1. Introduction

The epidemiological context of the coronavirus disease 2019 (COVID-19) pandemic has had wide-reaching impacts on all segments and sectors of society, imposing severe restrictions on the individuals’ participation in daily living activities, mobility and transport, on access to education, services and healthcare. This scenario is an unprecedented opportunity to speed up the development and implementation of innovative devices, biomedical solutions, and assistive technologies (AT) to facilitate persons with severe disabilities regarding their participation in daily life [1].

In recent years, the Brain-Computer Interface (BCI) application has been growing rapidly, establishing itself as an emerging technology able to translate human intentions into control signals and allow disabled people to interact with the external environment without any kinesthetic movement [1]. It has mainly targeted patients with neurological diseases such as amyotrophic lateral sclerosis (ALS), brainstem stroke, multiple sclerosis, and high spinal cord injury.

ALS is a progressive neurodegenerative disease that mainly affects motor neurons in the cerebral cortex, brainstem, and spinal cord. As the disease progresses, it led to a condition characterized by loss of ability in controlling the voluntary muscles. Subjects are aware of everything going around (the brain works properly). Still, they show limited (LIS: locked-in syndrome) or no motor response (CLIS: completely locked-in syndrome), meaning that the movement commands are not transmitted through the body limbs.

For these people unable to easily transmit their intentions to external devices using conventional interfaces such as a mouse or a keyboard, the development of brain-controlled systems could be the optimal solution to allow them to live their daily life [2,3]. Indeed, BCI is a useful tool to establish an additional communication channel between the subjects and external devices through users’ cerebral patterns. Thus, this approach can be efficiently used to improve their independence and facilitate participation in activities, thus enhancing overall well-being, reducing marginalization, and preventing impairments. In the pandemic context, in order to avoid any contamination risk, many public places have been forced to adopt specific systems and solutions to let people in and out. For subjects with a physical disability like those affected by ASL and for wheelchair users, this could mean having to use a longer path or having to deal with narrower halls. In addition, ALS patients’ management has become highly complicated [4] due to suspension or postponement of the outpatient follow-up visits. Several innovative solutions of telehealth, telemedicine [4,5], and remote monitoring systems [6] as well as new emerging technologies facilitating communication, mobility, and environment interaction/control have been proposed, but their full impact is yet to be determined [7].

The actual context has therefore strongly focused attention on the need to review the existing technologies and solutions for patients with severe disabilities already in place or under evaluation, as well as on the exigency to highlight their limitations, paving the way for future and helpful research in the BCI field.

More specifically, we identified a sub-area of interest in the BCI context that focuses on electroencephalography (EEG)-based BCIs, particularly those using motor-imagery (MI) data, for wheelchair movement and control.

EEG-based BCI has emerged as a technology with high translational potential owing to its desirable traits: direct measures of neural activity, portability, non-invasiveness, and inexpensiveness [8]. EEG-based BCI technologies in controlling mobile robots, particularly wheelchair systems, have been the subject of recent research interest. Several contributions have been published during the last decade to provide state-of-the-art wheelchairs driven by a brain-computer interface. Two of them [9,10] contain a survey, partially connected to this field, on brain-controlled mobile robots, describing the overall systems and the key techniques and the evaluation parameters of these robots. The other three articles [11,12,13] presented an extensive overview of current BCI-based wheelchair solutions. A recent paper [14] provides a detailed review of EEG signal processing in robot control (mobile robots and robotic arms), mainly based on non-invasive brain-computer interface systems.

Our paper aims to present the state-of-the-art applications of EEG-based BCIs, particularly those using motor-imagery (MI) data, to wheelchair movement and control in a real environment. Focusing on the applicability and feasibility of brain-controlled wheelchairs in the pandemic context and highlighting the need for easy usability required for disabled people, we considered studies that are based only on motor imagery EEG data and that tested the BCI approach on a real wheelchair, or at least a prototype, but not a simulator. The review presents a thorough examination of the different studies conducted since 2010, focusing on the algorithm analysis, features extraction, features selection, and classification techniques used, and wheelchair components and performance evaluation.

The rest of this paper is organized as follows: Section 2 describes more in detail about the methodology used for this review. Section 3 presents a synthetic overview of BCIs classifications and applications, identifying our area of interest more precisely. Section 4 focuses on applying MI EEG-based BCIs in wheelchair movement and control and summarizes the different existing solutions. Section 5 discusses algorithm analysis, features extraction, features selection, classification techniques, and software platforms used in the selected contributions, while Section 6 focuses on aspects related to performance evaluation criteria of brain-controlled wheelchair systems. Finally, Section 7 presents the main conclusions of this study and focuses on the primary challenges of biomedical research applied to people with severe disabilities.

## 2. Methodology for This Review

### 2.1. Search Strategy

This systematic review was conducted following the preferred reporting items for systematic reviews and meta-analyses [15]. A comprehensive literature search was conducted on 1 March 2021. The most common engineering and medical databases (IEEE Xplore, Pubmed, Science Direct and Scopus) were selected for research. The review was limited to texts published in English between 2010 and 2021, for which abstracts were available. Considering the scope of the systematic review, the specific keywords were defined. This structured search string was used to organize this paper: “motor imagery”-AND-“EEG-based” OR “electroencephalography-based”-AND-“BCI” OR “Brain-Computer Interface”-AND-“Wheelchair movement”-AND-“control”. To increase the likelihood that all the relevant studies were identified, additional articles identified through the reference list of previously retrieved articles were included.

### 2.2. Inclusion and Exclusion Criteria

Articles were considered for inclusion only if: (1) they described brain-computer interface systems based on motor imagery paradigms as mainly EEG acquisition modality; (2) they partially or totally demonstrate the feasibility, effectiveness, and applicability of MI EEG-based BCIs for wheelchair movement or control in real-world settings; (3) they described completed research.

The articles were also screened for the following exclusion criteria: (1) contributions that described BCI systems mainly based on face gestures or intentional blinks to control wheelchair; (2) studies that described only a simulated system or virtual environment and in which there is no reference to real wheelchair (or prototype) movement and control; (3) studies that presented a multimodal-mental approach, that is BCWs that are based on more than one type of EEG signal combined together (e.g., ERD/ERS, P300, and SSVEP) for their control. Exclusion criteria were also related to papers, books or book chapters, letters, review articles, editorials, and short communications.

### 2.3. Study Selection

Our work aims to present state-of-the-art applications of EEG-based BCIs using motor-imagery data to wheelchair movement and control. Since these interfaces’ target population are older people or patients with impaired motor abilities and considered our interest in assessing applicability in a daily context, only the studies that described a real wheelchair, or at least a prototype, but not a simulated system, are investigated and reported. A particular emphasis was given to studies that performed experiment evaluation of wheelchair navigation in a real-world environment.

**Figure 1:**
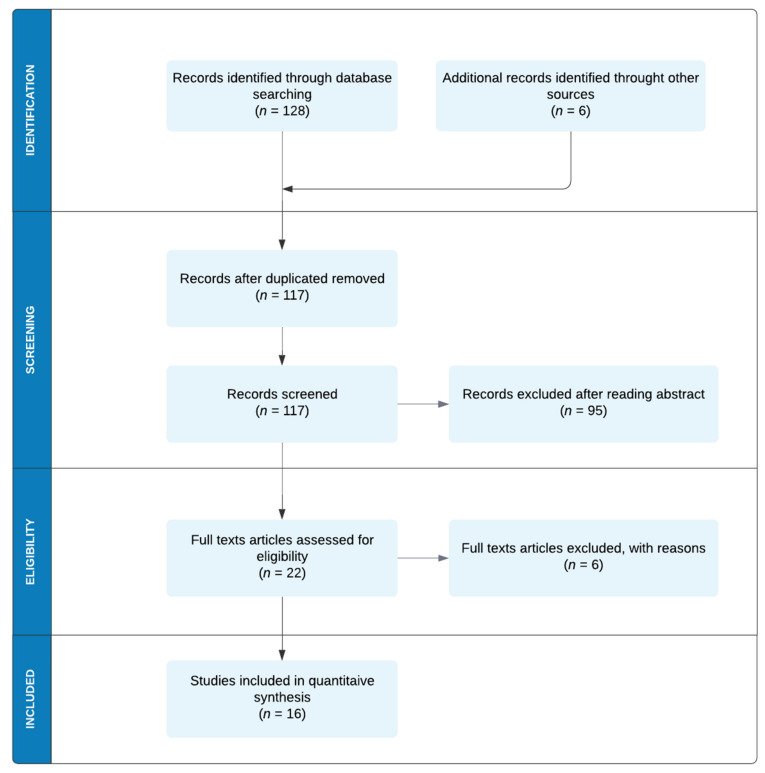
Flow chart of the search strategy and study selection according to PRISMA guidelines.

A total of 134 search results were identified through database searching and additional sources. After removing all duplicates, 117 studies underwent title and abstract screening, and the inclusion criteria were examined. The full texts of 22 papers assessed for eligibility were carefully analyzed. Three articles [16,17,18] were excluded due to the exclusion criteria (1), one contribution [19] due to the exclusion criteria (2), and two scientific results [20,21] due to the exclusion criteria (3). Finally, only 16 studies were included in the quantitative synthesis. The methodological approach is presented in Figure 1. For facilitating analysis and comparisons, we summarized all relevant BCI existing solutions and related system parameters in Table 1. In Section 4, we discussed each study, contextualizing the results in the BCI realm.

## 3. Brain–Computer Interfaces Classifications and Applications: A Synthetic Overview

Far from representing an exhaustive and detailed description of BCI systems’ main characteristics and classifications, discussed in depth in several contributions [37,38], this section aims to frame the area of interest of our review more precisely. According to their invasiveness, BCIs can be classified into invasive and non-invasive ones, depending on whether sensors used to measure brain activity penetrate the skin or not [39]. In invasive BCIs, cerebral signals are acquired inside the brain using electrodes located under the skull. The two invasive modalities mainly used in BCI research are intracortical recording and electrocorticography (ECoG).

We focused our research on the non-invasive BCIs in which brain signals are acquired using sensors placed on the scalp. Among various non-invasive brain-imaging methods often used to implement BCI systems (EEG, Magnetoencephalography (MEG), Positron emission tomography (PET), functional magnetic resonance imaging (fMRI), and Functional Near-Infrared Spectroscopy (fNIRS)), over the last couple of decades, EEG has been the most widely employed due to its desirable traits, namely non-invasiveness, portability, high temporal resolution, and a relatively low cost compared to other neuroimaging methodologies [37,40].

In recent years, up-and-coming practical applications of EEG-based BCI with several elaborately designed paradigms [41] are being evaluated [42,43,44,45,46,47]. Within EEG-based BCI paradigms, two groups can be roughly identified: exogenous (or evoked), which use external triggers (flickering LEDs or auditory beeps) to evoke discriminative brain patterns, and endogenous (or spontaneous), which use self-regulation of brainwaves without external stimuli [40].

Typical examples of exogenous BCI paradigms are the steady-state visual evoked potential (SSVEP) [42,43] and the P300 signal [44,45]. P300 [48,49] is a localized brain pattern response to an external attended visual, auditory, or tactile stimulus and is mainly measured in the parietal lobe. SSVEP [50,51] is a response to a visual stimulus at a frequency greater than 6 Hz, which can be primarily observed in the occipital area. On the other hand, Event-Related Desynchronization/Synchronization (ERD/ERS) changes, elicited during the performance of mental tasks (e.g., motor imagery, mental arithmetic, and mental rotation) [46,47], are representative of endogenous BCIs paradigms, as they do not use any external stimuli.

Since controlling a wheelchair requires that the visual channel remains dedicated to the maintenance of visual attention on the environment, the endogenous signals, particularly those elicited by MI, are to be preferred. Although learning to modulate endogenous signals requires more time for the users, MI paradigms present significant advantages that should not be overlooked for this review’s scope. Indeed, they do not require any external stimulation. In addition, they can be operated via free self-control and, consequently, they are particularly suitable and advantageous for the patients suffering from motor neuron diseases [40].

**Figure 2:**
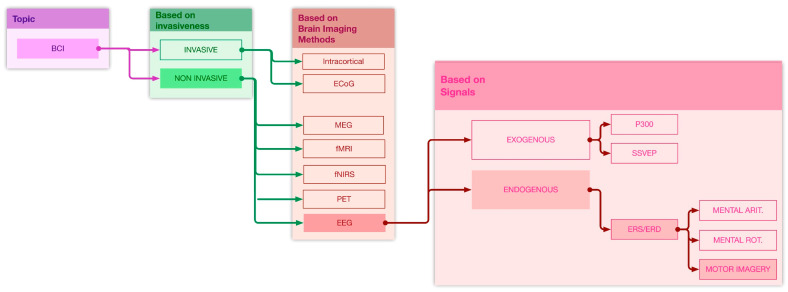
A synthetic overview of brain–computer Interface typologies and of biometric signals generally used in BCI systems. The path connecting the opaque colored blocks defines the sub-area of interest of the systematic review.

In light of these observations, EEG-based BCIs, particularly those using motor image paradigms applied to wheelchair apparatus control, represent the sub-area of interest of our review (Figure 2). Although they are outside the scope of this work, for completeness of discussion, other relevant uses of motor imagery BCI must be cited and analyzed. Indeed, in addition to managing wheelchair movement and control, a MI BCI has a wide range of applications, such as virtual reality, neurorehabilitation, and controlling robotic devices [52]. The scientific research on BCI technology has also been focused on other medical applications [52,53,54,55], with many BCIs intended for the replacement or restoration of central nervous system (CNS) functionality lost due to illnesses (such as amyotrophic lateral sclerosis and locked-in syndrome) or to trauma (such as spinal cord injury), and others focused on therapy and motor rehabilitation [38].

Since the neural mechanism involved in an MI-BCI system is connected to the motor function, these systems have been thoroughly evaluated and therefore taken into consideration for their potential applications in the fields of motor control, neurological rehabilitation training, and motor learning.

The use of brain–computer interface technology in detecting mental intent and controlling external robotic devices allowed to improve the lives of patients suffering from various neurological disorders. As described in depth in a recent comprehensive review of Aljalal et al. [14], although a robotic arm is a mechanical device, it has a certain number of degrees of freedom (DOF) and ends in a robotic hand, which gives it the functions similar to that of human arm. In this context, the purpose of EEG-based BCIs is to translate the signals generated by the patient’s mental tasks to allow the movement and control of a prosthetic limb, an orthosis, or an exoskeleton as an assistive device. Several exemplary applications of MI-EEG-based BCIs in robotic arms control can be found in [14,56,57].

In addition to neuroprosthetics, the use of MI-based BCIs attracted considerable interest also as a potential neurorehabilitation technique to restore motor function after stroke [38,58,59]. Indeed, the scientific interest in the use of robotics in rehabilitation scenario is increasing considerably due to the growing number of people requiring rehabilitation following problems such as stroke and, at the same time, to the insufficient number of therapists available to deliver rehabilitation protocols to patients [59]. The main objective of robotic systems in the rehabilitation field is to allow the robot, rather than the therapist, to guide the exercises provided for in the rehabilitation protocol, thus helping the patient to actively undertake a planned movement rather than the patient’s limb, which is passively moved [59]. Several studies involving BCI training in which motor imagery-related EEG activity is translated into movements of an exoskeleton have demonstrated improvements in clinical parameters of post-stroke motor recovery [60,61,62,63].

Another interesting application, rather than focusing on the machine learning aspects of MI BCI training, aims at corroborating the importance and efficacy of mutual (or co-adaptive) learning methodology as a critical factor for the success of motor imagery BCI in translation application. Co-adaptive approaches are recently increasing adopted as a training strategy [64,65,66,67,68] and require that the user and the embedded decoder engage in a mutual learning process [68]. In this context, the success of a BCI-user symbiotic system requires that users must learn to generate distinct brain responses for different mental tasks, while machine learning techniques to implement and adapt a model to the potentially changing brain patterns associated to these tasks [67]. This feature could lead to a BCI system able to succeed in a real-world scenario [68].

## 4. MI EEG-Based BCIs in Wheelchair Movement and Control: Literature Results

Over the last years, among various BCI applications, using the human brain in wheelchair movement and control is attracting widespread attention in the scientific community due to its flexibility and potential to help old and paralyzed individuals gain independence and potentially improve their quality of life. Since the first demonstrations of feasibility that the “human mind can control a wheelchair” [69,70], several protocols have been proposed, and a sophisticated algorithm has been implemented to extend the applications of EEG-based BCIs to wheelchair movement and control [12]. Following the definition given in [14], the wheelchair is classified as a mobile robot that can navigate two dimensions.

EEG-based wheelchair system refers to a type of brain–computer interfaces technology in which this specific mobile robot is controlled using electroencephalographic patterns collected from the human brain. This technological approach allows the subject to reach a particular target using only brain signals. Despite the enormous interest in implementing a brain-controlled wheelchair (BCW) that can improve disabled people autonomy allowing them to move through a real environment [11], the number of scientific contributions in the field is not very high due to the complexity of developing such an elaborate system [25].

In presenting the background of recent studies on wheelchair control through the acquisition of a user’s brain activity, the groups of Al-qaysi [12] and Fernández-Rodríguez [11] analyzed several BCW existing solutions: MI-based BCW [23,28,71], P300-based BCW [72,73,74,75,76], SSVEP-based BCW [77,78,79,80], and hybrid-based BCW [81,82]. Hybridization is a relatively new concept in the context of BCI, showing promising and interesting results in different domains, as it exploits the conjunction of different brain and body monitoring methods to achieve more accurate and comprehensive systems [83,84]. A simple, complete and highly accepted definition identifies the hybrid brain computer interface (hBCI) as “a system that combines two or more signals from different origins, including at least one input recorded directly from the brain” [85]. More specifically, a hybrid-based BCW is commonly identified as a system based on one EEG input combined with one or more channels (e.g., EEG, electromyography (EMG), electrooculography (EOG), or movement detection) to manage control and movement of a wheelchair. All these studies present a standard signal acquisition methodology (EEG) to control the system, but different structural elements: the specific signals used to implement the BCI system, the tasks to be performed by users, the number and type of commands available on the device, the modality of navigation, etc.

As mentioned above, our interest is to prove the feasibility and applicability of a brain-controlled wheelchair in a real environment considering, as the target population, patients with impaired motor abilities. For this reason, among the four EEG control signals models used to handle BCI wheelchairs, those based on motor-imagery task can be considered the most appropriate choice for achieving the intended purpose. Indeed, a motor-imagery paradigm does not rely on visual stimuli and does not interfere with navigation’s visual task, allowing the user to control the wheelchair spontaneously. The subject is not exposed to any stimulation, and thus there is no risk of fatigue. In addition, a brain-controlled wheelchair based on the motor imagery paradigm is more appropriate for use in an unknown environment, and several classes of identified motor imagery output can be directly transmitted into the directional control of a robotic wheelchair [36]. Finally, the use of MI when dealing with motor-disabled patients makes sense since this paradigm does not interfere with the patient’s residual capabilities, involving a part of the cortex that may have effectively become redundant [30].

This systematic review focuses on key issues related to non-invasive EEG-based BCIs that use motor imagery as the main paradigm applied to wheelchair movement and control.

### Existing Applications of MI EEG BCW

In this section, several existing applications of MI EEG-based BCIs for wheelchair movement and control are illustrated. These studies are carefully analyzed, and the main characteristics in terms of signal acquisition, preprocessing, feature extraction, and classifications methods as well as wheelchair performances evaluation are summarized in Table 1. The studies are tabulated in chronological order.

Tsui et al. [36] presented a simple two-class self-paced MI-based BCI for wheelchair control. With this system, the user was able to make path planning and fully control the wheelchair. Based on a laser range finder, an automatic obstacle avoidance system is integrated with the robotic wheelchair’s control mechanism. After practicing with the simulator, the system was tested online in the University of Essex’s robotic arena and the experiments were carried out with two subjects.

The work of Carrino et al. [35] proposed a user-friendly, self-paced BCI system that, using a commercial EEG headset and a motor imagery approach, allows the user to drive an electric wheelchair. Although the low-cost EEG device provided interesting results, the authors stated that it could hardly be used for self-paced systems in error sensitive contexts. Indeed, the system was tested directly on the wheelchair, and several problems occurred. More specifically, the classification process’s errors produced an unexpected behavior of the wheelchair and, thus, a strong perturbation for the user, concentrated in motor imagery tasks. Since this problem does not allow any kind of navigation for non-trained subjects, the test was finally performed involving one subject and using real gestures, less sensitive to emotional perturbations.

A novel wheelchair system controlled by EEG signals was constructed by Choi et al. [34], using effective signal processing methods to allow people paralyzed from the neck down to interact with society more freely. The implemented system was evaluated through experiments on controlling bars and avoiding obstacles using three subjects. The authors confirmed that the proposed wheelchair system demonstrated almost the same performance as a wheelchair controlled by a joystick.

In Li et al. [33], authors evaluated the feasibility of a BCI-based wheelchair, in which, users’ thoughts can steer without any additional involvements. In practical driving testing in a real environment, which involved three healthy participants, the system achieved a good performance, suggesting a potential application to people with disabilities in daily life. For future improvements in terms of usability of assistive wheelchair systems, the authors considered integrating infrared sensors and adopt other types of EEG signals.

The group of Carra [32] illustrated the development of a non-invasive experimental BCI system. The proposed approach generated commands to move a motorized wheelchair using portable and low-cost equipment and capture brain signals from the somatosensory cortex without the involvement of peripheral nerves and muscles. Experimental tests, which were performed in an uncontrolled environment and involved only one volunteer, showed promising results, thus enabling a possible future interface with real-life situations.

For designing a BCI system, Reshmi et al. [31] introduced five-class motor imagery EEG-based approach. The patterns acquired from the sensory-motor cortex are translated into a control signal to manage the directional movement of a wheelchair. Indeed, users’ movement intentions are classified according to the limb movements, and the results of patterns identification, tested by fifty control subjects, can be used as a command to move the wheelchair in the designated directions (right, left, forward, and backward) and to stop it.

Carlson et al. [30] proposed an asynchronous wheelchair system, integrated with robotics and computer vision techniques, which allows the subject to control the wheelchair by performing a motor-imagery task spontaneously. This group introduced the notion of shared control to integrate the user’s intelligence with the precise capabilities of a robotic wheelchair given the context of the surroundings. The authors demonstrated that several types of BCI wheelchair operators (four healthy subjects, new and experienced) could complete a navigation task successfully. Moreover, compared with an alternative P300-based system, the asynchronous MI approach gives users greater flexibility and authority over the actual trajectories driven. More specifically, the users can interact with the wheelchair spontaneously and can voluntary control the motion at all times, rather than having to wait for external cues. Besides, they can dynamically produce intuitive and smooth trajectories rather than relying on predefined routes, thus reducing the inactivity navigation time.

To overcome some of the limitations of several existing solutions, such as gaze dependence and unnecessary stops, Kim et al. [29] presented an MI-based brain-actuated wheelchair system using an extended five-command protocol. The presented wheelchair could be driven by the user in both smooth and right-angled turns. The system, validated by only one healthy subject, could be integrated with various robotic and computer vision sensors via additional channels in the network module, thus providing the user with appropriate feedback and improving safety. This approach can allow the user to cope with various environments, reaching a goal point with lower execution time.

In the study of Varona-Moya et al. [28], the authors tested the feasibility of driving a customized robotic wheelchair with an MI-based BCI system and the auditory cues to inform the subject of the available navigation command at every moment. To enable effective and autonomous wheelchair navigation, this group proposed an application interface that, based on a two-class sensorimotor rhythms-BCI paradigm, provided the user with four navigation commands. The results, obtained from a sample of three healthy naïve participants, suggested that this system seems to be an effective way of driving a robotic wheelchair autonomously and could provide locked-in patients with a better quality of life.

Swee et al. [27] proposed developing an electric wheelchair that can be directly controlled by the brain and that does not require any physical feedback as controlling input from the user. EEG signals, acquired with a commercial headset, are processed and converted into mental commands and a specific implemented algorithm transmitted out the controlling signals wirelessly to the electrical wheelchair. The authors anticipated that this system, tested by five healthy users, could give a new contribution to physically disabled people to regain their mobility.

Zhang et al. [26] demonstrated the effectiveness of a brain-controlled intelligent wheelchair that combines an MI (or alternatively P300)-based BCI and an automated navigation system. For the scope of this review, only the MI-based BCI solution was taken into account. The proposed wheelchair, tested by three healthy subjects, has several advantages: (i) it can adapt to changes in the environment; (ii) once the user selects a destination with the BCI, the system automatically navigates to it, allowing the workload reduction for the user; (iii) during the wheelchair navigation, the user can issue a stop command via the BCI.

To provide several navigation commands without worsening the system performance, a paradigm based on the discrimination of only two mental tasks to control the wheelchair is presented in the study of Ron-Angevin et al. [25]. Such a non-muscular control system has the peculiarity that it is embedded with an auditory interface that provides the user with four navigation commands. The authors suggested that this system, validated by seventeen healthy participants, could be an effective option to allow wheelchair displacement in a controlled environment for potential users with motor neuron diseases in the face of more extensive training.

The group of Al-Turabi [24] described the experience of developing a complete BCI system able to instruct a wheelchair to move to different directions using non-invasive EEG brain waves. Several machine-learning algorithms are used to classify human intention to control and move the wheelchair to the desired direction. In light of the experimental results, conducted involving only one neurologically health volunteer, the authors proposed their system to control other devices and hypothesized, as a future improvement, a cloud-based system to direct communication from the headset to the wheelchair.

In Yu et al. [23], the authors implemented an asynchronous control strategy in which the wheelchair commands are generated by a multi-step process based on sequential MI, without any external prompt information. Although the system was tested in seven healthy subjects, the preliminary experimental results demonstrated this navigation strategy’s potential applicability in enhancing the mobility of people with physical disabilities in a real environment.

Permana’s project purpose [22] was to control the wheelchair using a motor imagination-based BCI and a portable EEG device. In performing a preliminary experimental test using only MI patterns from a single data channel to trigger the wheelchair movement, the authors found some problems. Indeed, due to similarities in EEG patterns related to different motor paradigms, the classification for several wheelchair control signals failed. To overcome this limitation, authors added a new variable (eyes motion) as a differentiator of similar data, without obtaining evaluable results. In conclusions, Permana’s system, validated by five normal people, still needs to be developed and improved.

In a recent work of Xiong et al. [8], the authors made several important contributions to the state-of-the-art in BCIs. They proposed a wheelchair prototype that uses hand motor imagery and jaw clench data collected with a consumer-grade EEG system to generate four control commands, bridging the gap between the real-time classification of motor imagery and the use of a low-cost apparatus. Additionally, different automated driving features, a location tracker, and a heart-rate monitor have been integrated into the system to increase usability and safety. A pilot cohort of seven healthy volunteers were recruited to collect an MI training data set. Although future experiments and a consistent neurofeedback training procedure were required to validate their prototype, this system seems to get closer to the actual context’s needs and demands. The authors highlighted that this system’s clinical applications would largely depend on the motor abilities of the user (EMG toggle would be inaccessible to patients with more severe disabilities such as CLIS) and proposed, as a future piece of work, other integrative non-muscular signals, such as electrooculography (EOG).

## 5. MI-Based BCW Elements

**Figure 3:**
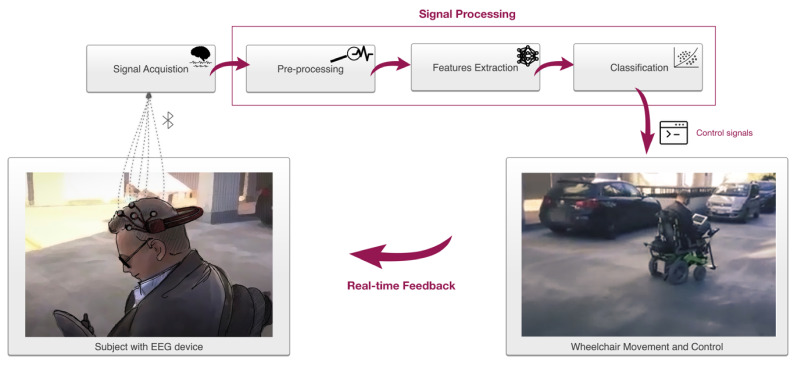
The general architecture of a brain-controlled wheelchair system and application example in a real environment. Reproduced with permission [86,87]. Graphics by [88].

In the design and implementation of a brain-based control wheelchair system, four stages are necessary to establish the communication between the human brain and the external device and to get a useful output to be used in controlling it: brain signals acquisition, preprocessing, features extraction from patterns and features classification. An example of brain-controlled wheelchair components and the system application in a real environment is illustrated in Figure 3.

A successful BCI system must be characterized by the best accuracy in extracting EEG features and classifying them. Indeed, since the presence of errors can cause the initiation of a wrong command that can lead to dangerous situations, a high classification rate and accuracy are required [18]. For this reason, features extraction and classification processes play a significant role. In this section, we discussed these four stages in more detail, together with published examples and in the light of summarized results. As integration, the software libraries primarily used in the collected studies were illustrated and analyzed.

### 5.1. Signal Acquisition

As mentioned above, this systematic review focuses on the non-invasive methods on which the applications of EEG-based brain-controlled wheelchair are based. Many EEG data acquisition devices are available in commerce, which vary in the number of channels, sampling rate, electrode connection type, headset preparation time, and price [14,89,90]. Our review results revealed that, in MI-based brain-controlled wheelchair applications, the most used devices to capture EEG signals are Epoc [91], produced by Emotiv Systems Inc., and the g.tec medical engineering products, such as gUSBamp.

The Emotiv Epoc is a portable, high-resolution EEG system with 14 dry electrodes designed to be quick and easy to fit, taking practical application measurements. Many BCI studies used EPOC to control or interact with machines in users’ environments [92], although the validity of Emotiv products in clinical research is still a matter of debate.

On the other hand, g.USBamp RESEARCH [93], a high-performance and high-accuracy 16 channel biosignal amplifier, was proposed by g.tec medical engineering to acquire and process physiological signals. g.USBamp has become a widely used standard for neurophysiological research, life sciences, neurofeedback, and the brain–computer interface approach.

Also, brain product EEG amplifiers [94], such as BrainAmp DC [23] and Acti-CHamp [28], were commonly used for a variety of practical uses in neurophysiological research. An overview of other used EEG signal recording devices is presented in Table 1, together with examples of associated brain-controlled wheelchairs.

### 5.2. Pre-Processing

Signal preprocessing is a non-trivial step required to clean data and remove any unwanted components (noise, artefacts, or interference) embedded within the EEG signals [14,37]. A proper preprocessing procedure produces an improvement in the signal quality and results in better feature separability and classification performance.

The most common methods applied in BCIs preprocessing and adopted in the summarized scientific contributions are frequency-domain filtering and spatial filtering. Bandpass filters [8,23,25,26,28,31,32], the primary attempts to attenuate artefacts in the measured EEG, and notch filters [24,34], used to remove the noise generated by the power line, are examples of frequency domain preprocessing solutions. However, only when the frequency bands of the signal do not overlap, these methods are effective.

A spatial filter is an alternative approach to increase the signal-to-noise ratio (SNR) of the brain signal. Typical examples of spatial filtering methods are Laplacian filtering [30,35], blind source separation (BSS) [34], common average reference (CAR) [29,33], autocorrelation (AC), canonical correlation analysis (CCA), independent component analysis (ICA), minimum energy combination (MEC), and principal component analysis (PCA). For real-time BCI applications, automatic methods and low computational cost are required. Recently emerging algorithms, such as independent vector analysis (IVA), a modified joint BSS approach (JBSS), a quadrature regression IVA (q-IVA), and the filter-bank-based supervised machine learning approach, introduced more effective artefact removal approaches, paving the way for innovative and helpful research in the BCI field [95]. A detailed description of the mentioned methods out of this review’s scope can be found in [14,37,38,96,97].

### 5.3. Feature Extraction

EEG features for MI BCI are related to both spectral and spatial domains. Although the feature extraction methods used in the selected studies are quite heterogeneous, analysis in the spatial domain using Common Spectral Patterns (CSP) resulted in being the general approach [24,26,29,33,34].

Being employed since 2000 to detect event-related desynchronizations [98], CSP filter is mentioned as an effective way to discriminate classes and is one of the most popular feature extraction methods in the BCI field [38]. Specifically, it is widely used for high recognition and low computational complexity. This method aims to transform EEG data into a new space, maximizing the variance of the (projected) signal from one class and simultaneously minimizing it for the other class. More in details, the Wavelet transform is applied to the preprocessed EEG data (represented as a matrix of size N × S, where N is the number of channels and S is the number of samples per channel). The output of the Wavelet transform is the input of the CSP algorithm. It is considered a strong technique in MI EEG processing since it enables the extraction of signal information from particular frequency bands. However, proper selection of the filtering frequency band dramatically affects the performance of CSP, and the optimal frequency band is typically subject-specific. Thus, it is difficult to select manually. The common spatial frequency subspace decomposition (CSFSD) method, adopted in Choi et al. [34] and used in Ramoser et al. [98], is a modified type of CSP that employs frequency and spatial filtering. The CSFSD aims to estimate spatial frequency filters corresponding to left and right movement imagination. The limit of the CSFSD is that it can be used only for classifying two groups of data. For example, in Choi et al. [34], the total set of CSFSD is summed to classify three data groups.

In other summarized BCW systems, the power spectral density (PSD) method is also adopted. PSD is the measure of how the power of a signal is distributed over frequency. Power spectral density estimation is performed through parametric or non-parametric methods. The former is based on the autoregressive model or the adaptive autoregressive model. The latter group includes Fast Fourier Transform (FFT)-based methods and variation of FFT, such as Welch’s method [96]. It is an efficient frequency domain-based feature widely used in motor imagery paradigms, but its performance may decrease seriously when applied to low SNR data.

### 5.4. Pattern Classification

Implementing a successful BCI approach requires that the system identifies several user brain activity patterns, extract from them the most significant features, and classify them with the best accuracy. The classification step converts the user’s intention into command signals for an output device (for example, a wheelchair).

Especially in third-party device control applications, where errors can lead to dangerous situations, high classification rate and accuracy are mandatory [18]. Although either regression or classification algorithms could be employed to achieve the goal above, the latter’s use is considered to be the most popular approach [99]. Among the numerous classification algorithms commonly used in BCI’s scientific context [37] and especially in brain-controlled mobile robots [14], the SVM-based and LDA-based approaches predominate in the studies of this review.

LDA is an effective statistical technique used as a well-known binary classification method for EEG data. It is employed to identify the linear combinations of feature vectors that characterize the corresponding signal. This method projects data in a new space and uses a hyperplane to distinguish different classes, minimizing the variance within a class and maximizing the variance between classes [14]. Thanks to its satisfactory performance, very low computational cost, easy use, and no requirement of extensive pretraining, an LDA classifier is to be preferred in various BCI systems, for example, in a motor imagery-based approach. However, its linearity can cause performance degradation and poor results in a few circumstances containing complex large non-linear EEG data.

On the other hand, SVM, first proposed by Vapnik et al. [100], is a supervised learning algorithm used to solve binary classification problems by creating a linear optimal hyperplane. To perform a classification process for a given set of training data, SVM constructs a hyperplane model in a multidimensional space that separates the patterns belonging to the different classes by the widest margin [14]. As reported in Padfield et al. [38], the SVM classifier resulted in higher performance when compared to LDA and regression algorithms.

In addition to LDA and SVM classification solutions, artificial neural network (ANN) and k-nearest neighbor (k-NN) [101] are also adopted. The element that characterizes the neural network (NN) lies in their special ability to extract patterns and identify trends challenging to find, either by humans or by computerized techniques. A trained NN algorithm, one of the fundamental tools utilized in machine learning, can be recognized as an “expert” in performing classification of information that it has been provided to analyze [37]. An ANN is a multi-class classifier, widely used in the BCI field, based on a brain-inspired information system that simulates and replicates the process of human cognition [14]. KNN is a supervised learning algorithm that can be used to classify between two or more patterns. It is based on the concept that features related to different patterns will result in different clusters in the features space, while similar patterns will form convergent or similar clusters. The BCI community does not seem to widely use this method due to its sensitivity to the curse-of-dimensionality, which causes it to fail in several experiments.

### 5.5. Software Platforms

Several commercial software platforms, toolboxes, and frameworks were adopted to implement the necessary steps for EEG signal processing, such as filtering, artefact correction, feature extraction, and classification. An overview of the most widely-used BCI platforms is presented, together with examples of associated brain-controlled wheelchairs (Table 1). In-depth and detailed discussions of the technical characteristics of all platforms are reported in other studies [102,103], and as such, is not the main purpose of our work. However, we believe it is appropriate to list and highlight, for each of them, several features that could be essential in wheelchair control application, such as intended target user group, availability on different operating systems, licenses, programming languages involved, supported devices, performance, and so on. Typically, the target user group of these frameworks consists either of BCI developers, BCI users, or both [102]. As far as licenses are concerned, some platforms are released under popular open source ones (such as the GNU General Public License [104]), which allow everyone to apply changes and redistribute the source code [102]. Moreover, frameworks can be cross-platform (i.e., deployed on several different operating systems) or restricted to either a specific operating system and/or require commercial software.

We have identified some major platforms (OpenVibe, OpenBCI GUI, MATLAB/Simulink, and LABVIEW) [105,106,107], that were used in our review results, and other additional ones (Wyrm, BCI2000, BCILAB and Gumpy) [103,108,109,110,111,112,113,114], which, although not used in the aforementioned BCW systems, are also specifically aimed at BCI development and therefore worth mentioning.

OpenVibe [105], developed by the French National Institute for Research in Computer Science and Control (INRIA), is a free and open-source platform to design, test, and use BCI systems in both real and virtual environments. Adopted in Carrino et al. [35], it can be used for real-time processing and analysis of brain signals (acquire, filter, process, classify, and visualize data) due to its modularity features, portability, and flexibility. OpenViBE is designed for different types of users, including researchers, developers, and clinicians. Indeed, its easy-to-use graphical user interface is also suitable for non-programmers. This software platform supports several acquisition devices such as EEG or MEG amplifiers and can be easily integrated with high-level applications such as virtual reality (VR) applications. OpenViBE is licensed under the GNU Affero General Public License v3.0 (AGPL-3) and is officially available for Microsoft Windows and Linux (Ubuntu and Fedora) platforms. The evaluation of the platform performances allows to conclude that OpenViBE could prove a valuable and useful tool to design innovative BCI-based interaction devices for VR and confirms its suitability for real-time applications [102].

OpenBCI GUI [106], used in Xiong et al. [8], is an OpenBCI software that offers a powerful tool for visualizing, recording, and streaming data from the different OpenBCI Boards. Data can be transmitted in live-time to third-party software such as MATLAB. As a drawback, this GUI does not provide the possibility of acquiring data under a particular BCI paradigm nor does it allow for the on-line process of the biosignals [115]. The OpenBCI GUI is provided under the MIT License and is free to modify or adapt to custom setup. In addition, it will run as a native application on MacOs X, Windows, and Linux.

Commercial high-level platforms (MATLAB, Simulink or LabVIEW) have been used in several real-time BCI demonstrations. MATLAB (MARrix LABoratory, The MathWorks, Inc., Natick, MA, USA) is also a powerful tool for researchers to test models and algorithms in the BCI field, benefiting from resourceful toolboxes and an easy implementation process [107]. It is configured with a commercial programming language for numerical computing that supports Linux, Windows, and MacOs X. Sophisticated algorithms for specific application domains can be implemented in MATLAB or a block-diagram can be developed using Simulink, an interactive environment for modeling, analyzing, and testing dynamic systems. Most of the summarized studies adopted this commercial platform because of its ease of use, expansive functionality, and its suitability for developing, prototyping, testing, and evaluating new algorithms, as well as for real-time and online processing methods and applications.

LabVIEW (Laboratory Virtual Instrument Engineering Workbench-National Instruments, Inc, Austin, TX, USA) is a high-level multiplatform graphical development environment also used in our review results to implement a brain-controlled wheelchair [28]. It can generally run on Windows and, depending on version used and with limited functionality, on Mac OS X and Linux. The evaluation of some platform performances, which are the ability to interface with external instrumentation together with the ability to use data acquisition modules for third-part biosignal acquisition systems, allows us to confirm that LabVIEW was successfully adopted within the BCI community.

Although not used in any of the studies summarized in this review, it is worth mentioning, within the brain-controlled wheelchair research, other open-source BCI platforms: Wyrm, BCI2000, BCILAB, and Gumpy.

Wyrm [108,109] is an open-source Python-based BCI package applicable to a broad range of neuroscientific problems. The toolbox offers several functionalities to analyze and visualize neurophysiological data in offline processing and real-time settings, like an online BCI application. More specifically, it implements a wide range of different algorithms, including standard signal processing algorithms, advanced filtering algorithms (like the CSP), analysis methods (like single-trial analysis), multivariate pattern analysis (MVPA), machine learning algorithms (like the LDA), and many more. The whole system runs on all major operating systems and is licensed under the terms of the MIT license. Authors confirmed that Wyrm is capable of performing offline and online experiments, and that all functions of the toolbox are carefully tested for accuracy and profiled for speed, allowing to solve the necessary computations very efficiently.

BCI2000 [110,111] is an open-source C++ based software developed in 2000 for real-time BCI systems application. It includes stimulus presentation functionality and provides the data acquisition and signal processing modules. Specifically, BCI2000 supports 19 different data acquisition systems by different manufacturers, including all major digital EEG amplifiers. Still, some important methods (e.g., discrete wavelet transform) and some classification techniques (e.g., deep learning) are not embedded. BCI2000 is available under the GNU General Public License (GPL) v3 and runs on multiple platforms, including Windows and Mac OS X, though it is currently fully tested and supported on Windows only. Since this software does not directly support other programming languages such as Matlab or Python, it is not easy to extend or integrate it with other toolboxes [103]. Despite the aforementioned limitations, BCI2000 is adopted in many studies in the fields related to BCI research [102] and is also supporting the only existing long-term in-home application of BCI technology for people who are severely disabled [116].

BCILAB [112,113], developed by “Swartz Center for Computational Neuroscience” (SCCN) and distributed free to help researchers in processing signal with Matlab, is among the earliest publicly available software packages for research purposes in the BCI community. Because of its MATLAB foundation, the major strength of the toolbox lies in implementing rapid prototyping, offline performance evaluation of new BCI applications, and real time testing in the same computational framework. BCILAB can boast of an easily extensible collection of currently over 100 methods from the literature (including signal processing, machine learning, and BCI-specific methods). BCILAB supports Windows, Linux, and Mac systems.

Gumpy [103,114] provides state-of-the-art algorithms, signal processing methods, and classification approaches that the scientific community has employed over the last 20 years. It is designed for a hybrid brain–computer interface and is implemented to chart a route ahead for new BCI improvements [103]. It is widely used by machine learning compilers, engineers, and neuroscientists. It is an open-source, easy-to-use, robust, and powerful Python toolbox suitable for EEG and EMG bio signal analysis, visualization, real-time streaming and decoding. More importantly, in addition to classical machine learning algorithms, Gumpy includes different deep learning models such as deep convolutional neural networks (CNN) [117], recurrent convolutional neural networks (RCNN), and Long Short-Term Memory (LSTM) [118], which can be used to classify sensory-motor rhythms from EEG signals. Those approaches have hitherto been rarely investigated in a BCI context and it seems that no existing BCI software integrates similar techniques [103]. Gumpy’s source code is released under the MIT license and is supported on Linux, Windows, and Mac OS X.

In light of our overview which also reports currently available platforms and frameworks for developing and deploying MI-BCW systems, we can conclude that, while some platforms offer a great number of features (for example, BCI2000, OpenViBE, and Gumpy), each solution presents its unique features and benefits. It is important to note that the combination of MATLAB and Simulink is probably one of the most popular commercial general-purpose platforms for developing brain controlled wheelchair applications. Indeed, many scientific research groups prefer to develop their own MATLAB-centered solutions for biosignal acquisition and processing, adapting them on different requirements and prospective users.

## 6. MI-BCWs Performance Evaluation

The performance of a designed brain-controlled wheelchair has a fundamental importance and should be quantified while navigating a predefined set of common obstacles. Achieving high performance in MI-based BCI is a challenge that researchers have been working on for years as it increases the responsiveness of the device, prevents user frustration, and improve the user’s experience.

Several performance evaluation criteria were used by researchers, as per their convenience. However, researchers have no standard performance metrics that could be widely adhered to facilitate comparisons between brain–computer systems. The results of our review show that in most of the works, information relating to performance evaluation and the metrics used are often missing or poorly described. However, we believe it is appropriate to report, where available, some details on how the BCW’s performance assessment was achieved.

In Tsui et al. [36], for the experimental test in an arena environment, the task was to drive the robotic wheelchair from the “start” position to the “target” position without collisions with obstacles placed in the room. Based on the information provided by the authors, performance was evaluated by the average time to finish a run (108.75 s for subject 1 and 114.75 s for subject 2) and the number of interactions (executed commands) required to reach all targets (average: 5.38 interactions/min for subject 1 and 4.58 interactions/min for subject 2).

In Choi’s study [34], two types of experiments were conducted. In the first, the subject controlled bars on a monitor, following an arrow, by imaginary movement. In this experiment, two subjects achieved a 95.00% success rate, and the one had a 91.66% success rate. In the second experiment, the subjects drove a wheelchair on a figure of eight course while avoiding two obstacles and they were instructed to reach the original position in the shortest distance and time. Success was defined as the subject returning to the original position from the starting position without colliding against any obstacle or the wall. Failure was defined as the subject touching any obstacle or the wall. In this obstacle avoidance experiment, all three subjects achieved over 90% success rate.

Li et al. [33] evaluated the BCW system in terms of accuracies and practical running testing in a real environment. In the first part of the protocol, the system recognizes user’s movement intentions according to changes in spectral power relevant to user’s mental tasks (corresponding to left, right, and feet motor imagery). Trial accuracy was obtained by counting the number of trials classified correctly for each participant, reaching an average value of 82.56%. In the practical driving testing, in which the participant was required to steer the wheelchair moving along a specific path without hitting the obstacles (that are chairs), a good performances in terms of smooth movement and obstacle avoidance was observed.

The test conducted by Carra et al. [32] consisted of a preliminary procedure in which a specific number of tracks (imaginary movement of the foot (front arrow) and right hand (right arrow)) are presented to the volunteer who becomes familiar with the experiment. The collected data are used for classifier training and to obtain the specific parameters of the volunteer. Afterwards, the first series of corresponding route stimuli was presented. Wheelchair moves in direction to the stimulus only if the classification result of the corresponding track is correct, while remains standing in place in the opposite case. The subject must complete a route of seven predetermined positions three times, with the minimum possible tracks. The hit rates for volunteer in each series were evaluated, with an average value of 65.7%.

In Carlson et al. [30], the subjects were instructed to perform an online BCI session (the wheelchair remained stationary), after which they were given 15–30 min to familiarize themselves with driving the wheelchair using each of the control conditions: a two-button manual input, which served as a benchmark, and the BCI system. The task was to enter a large open-plan room, navigate to two different tables while avoiding obstacles, passing through narrow openings and finishing by reaching a second doorway exit of the room. A good level of control was achieved in the stationary online BCI session with an average accuracy of 95% on all subjects, as well as in the driving task, completed successfully and without collisions.

Varona-Moya’s experimental procedure [28] consists of a training schedule and a robotic wheelchair navigation phase, in turn divided into two tasks. The first robotic navigation task consisted of driving the real wheelchair from the starting point to the goal using the audio-cued interface only. The second robotic navigation task consisted of going back along that path, i.e., to return to the starting point. System performance information is not provided in the text. For the results, authors confirmed that all participants were able to perform at least one robotic wheelchair navigation task via our BCI system. In addition: (i) the minimum time lapse for the first and the second robotic navigation task was 4 min 38 s and 5 min, respectively, and (ii) the second task required the same number of selections as the first task plus two extra ones (turn 90° to the right or to the left).

Using Emotiv EPOC headset in the system proposed by Swee et al. [27], the brainwaves in EEG form were translated into the metrics (facial expression, performance metrics, and mental commands) by means of different detection tools. More specifically, the mental command detection suite is used to interpret the user’s mental commands (push, pull, left, and right) in order to control the electrical wheelchair movement. The testing result for mental commands, involving five users, showed that the processed EEG data provide up to 90% of accuracy.

To validate the effectiveness of the intelligent wheelchair, Zhang et al. [26] conducted an experiment (Exp. 1 for the MI-based BCI) that involved two different environments (Scenario A and the complex Scenario B). In Scenario A, the subjects were required to consecutively perform three tasks in a room equipped with a few pieces of furniture. Authors validated the feasibility of the wheelchair system in a relatively complex home environment, namely, Scenario B (with more obstacles than Scenario A). More specifically, to evaluate Zhang’s intelligent wheelchair system, several performance metrics were adopted: concentration time (CT), concentration time for each selection (CTFES), false destination selection (FS), response time (RT), success rate (SR), error distance (ED), and false activation rate (FA) [26]. For experiment 1 (MI-based BCI) and the more complex home environment (Scenario B), the average performance indices are: CT: 23.8 s; CTFES: 4.3 s; FS: 0; SR: 94.7%; ED: 9.5 s; and FA: 0 (see Tables 1 and 2 in [26] for additional information).

The experimental procedure used in the study of Ron-Angevin et al. [25] involved a total of three sessions: (i) an initial calibration session, (ii) a navigation session in a VE (control of a virtual wheelchair), and finally, (iii) a navigation session in a real environment with the BCW. The real experimental trial, which we are most interested in, consisted of two navigation tasks: to drive the real wheelchair from the starting point to the goal (task 1) and to return along the same path to the starting point (task 2). Referring to the performance evaluation during navigation, the following metrics are used: recall (user’s ability to select the desired command); specificity (user’s ability to avoid unwanted commands; precision (which of the user’s selections are correct); Negative Predictive Value (NPV) (which of the users’ non-selections are correct); and accuracy (level of overall performance). On the real wheelchair control session, subjects achieved a medium accuracy level above 0.83 (see Table 4 in [25] for additional results).

Al-Turabi et al. [24] performed experimental tests comparing acquired EEG data, measured with a commercial device, while the volunteer thought for four seconds about each direction (left, right, forward, and backward), with the EEG reference data. If the reference data is smaller than the current data, the control signal is to stop the wheelchair (the user is not focusing in any direction). Otherwise, mu and beta frequency bands were extracted and given as input of machine learning algorithm. Several algorithms (SVM, KNN, and ANN) were tested to predict the output to be transferred wirelessly to the wheelchair and control it into the different directions. However, SVM algorithm showed the highest accuracy with 79.2%.

The experimental procedures adopted in Yu et al. [23] consist of offline training (for calibration of the classifiers’ parameters), simulated experiments, and online wheelchair navigation experiments. The online experiments were conducted to evaluate the overall control performance of the proposed wheelchair navigation strategy in a real-world indoor environment. The subjects were required to navigate the wheelchair from the starting point to the destination position following the pre-established route, while passing through six waypoints and avoiding obstacles. The authors measured the following performances metrics: tasks accomplished (times of accomplishment of the navigation experiment); time taken (the average time to accomplish each task); waypoint missed (number of waypoints missed); commands taken (number of commands used to accomplish each experiment); distances travelled (distances travelled to accomplish each experiment); angle explored (total turning angle to accomplish an experiment); and collisions (number of collisions with the obstacles). In summary (see Table 3 in [23] for more details), the seven subjects accomplished 99 of the 105 experimental trials and the success rate was 94.2%.

MILO system, described in Xiong et al. [8], implemented a novel user interface to allow its user to switch between fully autonomous driving (wheelchair can move roughly forward through its environment while automatically avoiding objects in its path) and a brain-controlled driving mode (control requires inferring left and right imagined hand movement in real-time with machine learning algorithms). Experimental protocol was developed to collect MI data for three conditions: (1) imagined left hand movement, (2) imagined right hand movement, and (3) rest, where participants were asked to not imagine moving either hand. Experiments allowed to decode the three target states based on µ-wave power. Using a logistic regression classifier, only two scalp electrodes and a two second window size, a mean subject accuracy of 60 ± 5% is achieved.

Unfortunately, no in-depth or confusing information is given on this issue in the study of Carrino et al. [35] as well as in Kim et al. [29], Reshmi et al. [31], and Permana et al. [22].

The results of our review show that the scientific community interested in the field of BCI should make a greater effort in identifying standard performance metrics that could facilitate comparisons between BCW systems.

## 7. Conclusions

Our scientific research group’s interest has been focused for years on the development of innovative technological solutions and assistive systems designed to preserve communication and interaction with the external world in people with ALS [6,86,87,119,120,121,122,123].

The pandemic emergency of COVID-19 has shed light on the needs of people with severe disabilities regarding their participation in daily living activities, mobility and transport, on access to education, services, and healthcare. Therefore, in this context, it was necessary to highlight the limitations of current biomedical instrumentations applied to people with severe disabilities to pave the way for future and helpful research in the BCI field. This systematic literature review aims to prove the feasibility and applicability of a brain-controlled wheelchair in a real environment considering, as the target population, patients with impaired motor abilities.

Wheelchairs are among the most appropriate equipment that can promote mobility and improve people’s autonomy, providing valuable aid, particularly to the elderly and people with physical impairments, to move through a real environment and to do daily routines and tasks with ease.

In implementing an efficient brain-controlled wheelchair, three main challenges need to be considered: (i) system control is multi-objective, including numerous and different commands (start and stop, direction and speed). The system based on a navigation approach with a specific mental task can offer only a few navigation commands. On the other hand, a high number of cognitive tasks increases the number of navigations commands, but it can worsen the BCI system’s performance. Producing numerous control signals is challenging for an EEG-based BCI and making an accurate control command is a time-consuming process. (ii) The efficacy and the performance of a BCI system largely depend on the user, who often fails to perform the MI required to produce direction control signals. (iii) Continuous control of wheelchair navigation may produce a large mental workload for the user, especially for disabled people.

Despite the enormous interest in implementing a brain-controlled wheelchair, the current solutions do not seem to fully satisfy the demands in today’s context, mainly due to the complexity of developing such an elaborate system. Although innovative technological solutions of considerable importance have been implemented in MI-BCI research, some critical issues still need to be resolved [97]. Firstly, as most of the published studies are based on synchronized MI-BCI in offline modality, there is a need to give more prominence to online BCI studies. In addition, the performance of a designed brain-controlled wheelchair should be quantified while navigating a predefined set of common obstacles. Several performance evaluation metrics were used by researchers, as per their convenience. However, researchers have no standard performance metrics that could be widely adhered to facilitate comparisons between brain–computer systems. Improving the performance of BCW is still a critical issue even after two decades of research. Taking advantage of sophisticated algorithms’ availability, future research in MI-BCI should concentrate more on reducing long calibration, on increasing the number of commands without, however, producing a large mental workload for the user. BCI illiteracy, reported in [97] as the users’ inability to produce required oscillatory pattern during motor imagery paradigm, leads to poor performance of MI-BCI. The current trend of researchers is to predict whether a user falls under BCI illiterate category or not and to use this information to improve the implementation of an optimal algorithm for decoding MI and design a better training protocol for enhancing user skills.

Several technical suggestions can be considered for the potential improvements of BCWs. Although some MI-BCI wheelchairs also include sensors for navigation aids, the results are limited. Specifically, the experiments were mostly done with healthy subjects and in controlled environments. Regarding this first point, it is necessary to understand whether the performance of disabled users is comparable to that of healthy ones. The possibility of the use of environmental control systems by people with severe disabilities has been investigated in very few studies. In non-structured environments, a complete control system with navigation components, including mapping, location, route planning, and obstacle avoidance, is needed. Moreover, to improve the safety of disabled people in the use of wheelchairs, even in unstructured environments, it could be useful to investigate the possibility of integrating telemonitoring systems. Second, existing BCIs offer rather poor ITR (Information Transfer Rate), the widely used evaluation metric for command BCI systems. The problem of poor information transfer rate of BCIs and its effect on reducing the commands user, restricts BCI utilization for locked-in persons. Therefore, future research should focus on increasing the ITR of BCI systems. Finally, in order to improve the classification performances, moving from conventional machine learning models to deep learning approaches could be the optimal solution.

In summary, compared to several contributions published during the last decade to provide state-of-the-art wheelchairs driven by a brain-computer interface [9,10,11,12,13,14], our review presents strengths and novelties as it aims:

- to define the sub-area of interest in BCI context, rather than proving a wide overview of brain–computer interface typologies and applications;
- to present the state-of-the-art applications of EEG-based BCIs, particularly those using motor-imagery data, to wheelchair movement and control in a real environment;
- to highlight the need for easy usability required for disabled people and to focus the attention on the applicability and feasibility of brain-controlled wheelchair in a real context;
- to analyze MI EEG-based BCIs applied to wheelchair movement and control, not only in terms of algorithm analysis, features extraction, features selection, classification techniques, and software used, but also adding information about wheelchair type and components, obstacle avoidance systems, and wheelchair performances evaluation;
- to make assumption and provide suggestions on potential improvements of these devices.

In conclusion, we hope the results provided in this paper will highlight the limitations of current biomedical instrumentations applied to people with severe disabilities and bring focus to innovative research topics.

## Figures and Tables

**Table 1:** Brain-controlled interface studies in wheelchair movement and control applications.

Reference	MI Paradigm	Types of Control Command	EEG System	Additional Biosignals Acquisition	n° of EEG Electrodes	EEG Sample Frequency (Hz)	EEG Features Extraction	Classification Algorithm	Context and Duration of the Experimental Tests	n° of Users	Performance ^§^	Wheelchair Type and Components	Obstacle Avoidance System	Software
Xiong et al., 2019 [8]	LH RH Jaw Clench	Left Right Forward Stop	OpenBCI’s Cyton Biosensing 32-bit board (also used for EMG signal), (OpenBCI, New York, NY, USA)	EMG ECG US Location	4: C1, C2, C3, C4	250	PSD	Logistic Regression	INDOOR (Office/Laboratory) Average duration: 5 min (run) * 4–19 (n° of runs) = 20–95 min	7 CTR	Mean subject accuracy: 60 ± 5%	Modified version of commercially available Orthofab Oasis 2008 wheelchair (Orthofab, Anjou, QC, Canada) with components: n° 2 commercial-grade 40A, 12 V PWM controllers connected to an Arduino Uno. Project: MILO: Mind Controlled Locomotive	n° 4 consumer-grade ultrasonic sensors	OpenBCI Graphical User Interface (GUI) Pyton Javascript
Permana et al., 2019 [22]	MI and eye motion -Think moving forward -Think moving backward -Think moving backward while continually move the eyes -Think moving forward while continually move the eyes -Default (motionless)	Move forward Move backward Turn left Turn right Default (motionless)	Neurosky Mindwave Mobile2	NO	1: Fp1	512	For MI: eSense score For eyes-motion: high alpha	n.d.	INDOOR (Office/Laboratory) Average duration: 5 min	5 CTR	Success rate range: 46, 67–82.22%	Modified version of JRWD 501 electric wheelchair	NO	Matlab
Yu et al., 2018 [23]	LH RH IDLE STATE	Move forward Turn left Turn right Accelerate Decelerating Stopping	BrainAmp DC, (Brain Products, GmbH, Germany)	NO	31: F3, F1, Fz, F2, F4, FC5, FC3, FC1, FCz, FC2, FC4, FC6, C5, C3, C1, Cz, C2, C4, C6, CP5, CP3, CP1, CPz, CP2, CP4, CP6, P3, P1, Pz, P2, P4	250	Multi CSP	LDA	INDOOR (Office/Laboratory) Average duration: Offline training: 8 s (trial) * 15 (n° of trials) = 2 min per mental task + 5 min (rest period) Online wheelchair navigation experiment (navigation time): 2106.4 s.	7 CTR	Accuracy: >85% Success rate: 94.2%	Wheelchair prototype: a chair and an omnidirectional moving vehicle	NO	n.d.
Al-Turabi et al., 2018 [24]	Imagine visually moving a pen	Forward, Backward Right Left	Emotiv Epoc	NO	14 (+2 ref): AF3; F7; F3; FC5; T7; P7; O1; O2; P8; T8; FC6; F4; F8; AF4 (+CMS and DRL)	128	PSD	SVM KNN ANN	INDOOR (Office/Laboratory) Average duration: n.d.	1 CTR	Accuracy: 70.8–79.2%	Wheelchair prototype	Ultrasonic sensor	Matlab
Ron-Angevin et al., 2017 [25]	RH IDLE STATE	Move forward Move backward, Turn right Turn left	Acti-CHamp amplifier (Brain Products GmbH, Munich, Germany)	NO	9: C3, F3, P3, T7, Cz, C4, F4, P4, T8.	200	Average signal power	LDA	INDOOR (Laboratory/University room) Average duration: Calibration session: 30 min Navigation session in a VE: 5–10 min Navigation session in a real environment with the BCW: 5–10 min.	17 CTR	Medium accuracy: 83%	Customized Invacare Mistral3 electric wheelchair	-n° 11 ultrasonic rangefinders SRF08 -n° 2 magnetic rotary encoders AS5048	Matlab
Zhang et al., 2016 * [26]	RH LH	Turn right Turn left Stop	EEG-cap (Compumedics, Neuroscan Inc., Abbotsford, Australia) EEG-amplified (NuAmps, Neuroscan)	NO	15: FC3, FCz, FC4, C3, Cz, C4, CP3, CPz, CP4, P3, Pz, P4, O1, Oz, O2	250	CSP	SVM	INDOOR (room/home environment) Average duration (time to complete a destination selection using the MI-based BCI): -24.3 s (Scenario A) -23.8 s (Scenario B)	3 CTR (MI-based BCI experiment)	Success rate: 94.7 ± 2.3%	Mid-wheel drive model 888WNLL, Pihsiang Machinery MFG. Co. Ltd., Taiwan, with sensors: - n° 1 laser range finder (SICK LMS 111) - n° 2 encoders, which are attached to the central driving wheels	n° 2 webcams n° 3 ultrasonic sensors	GUI
Swee et al., 2016 [27]	PUSH, PULL, LEFT, RIGHT	Forward Backward Left Right	Emotiv Epoc	NO	14 (+2 ref): AF3; F7; F3; FC5; T7; P7; O1; O2; P8; T8; FC6; F4; F8; AF4 (+CMS and DRL)	128	n.d.	n.d.	INDOOR (Office/Laboratory)	5 CTR	Accuracy: <90%	Wheelchair Prototype with components: Scooter motors DC 24 V ATmega328P microcontroller Arduino Uno microcontroller board Bluetooth Hc-06 module	NO	Emotiv API Arduino IDE
Varona-Moya et al., 2015 [28]	RH RELAX	Move forward Turn right Move backward Turn left	actiCHamp amplifier (Brain Products GmbH, Munich, Germany)	NO	9: F3, F4, T7, T8, C3, C4, P3, P4, Cz	200	PSD	LDA	INDOOR (Private room in the school) Average duration: Training schedule: 30 min (first phase) + 15 min (second phase) + 20 min (third phase) Robotic wheelchair navigation tasks (minimum time lapse): -4 min 38 s (for task 1) -5 min (for task 2)	3 CTR	n.d.	Customized Invacare “Mistral3” electric wheelchair	n° 11 SRF08 ultrasonic range finders (i.e., sonars) allowed to create a real-time discrete grid map of the area surrounding the wheelchair. n° 2 AS5048 magnetic rotary encoders were attached to the wheelchair’s driving wheels to carry out the odometry and thus compute the wheelchair’s heading at every moment.	Matlab
Kim et al., 2013 [29]	LH RH F F-LH F-RH	Left Right Forward Left-diagonal Right- diagonal	g.tec system (an EEG cap and a gUSBamp amplifier)	NO	16	256	OVR CSP	LDA OVR LDA	INDOOR (Office) Average duration: n.d.	1 CTR	n.d.	Electric wheelchair (K2 POWER model of WHEELOPIA), with components: n° 2 permanent magnet DC brushed motors (pMDC motors: 24 V at 320 W). n° 1 micro controller unit (MCV, Atmega128).	NO	Simulink Matlab
Carlson et al., 2013 [30]	RH LH F	Turn right Turn left Keep going straight	EEG device (model n.d.)	NO	16: Fz, FC3, FC1, FCz, FC2, FC4, C3, C1, Cz, C2, C4, CP3, CP1, CPz, CP2, CP4	512	PSD	Gaussian classifier	INDOOR (Office/Laboratory) Average duration: Online BCI session: 4–5 min Driving task: 15–30 min	4 CTR	Average accuracy: 95%	Modified version of commercial mid-wheel drive model by Invacare Corporation (TDX SP2)	n° 10 close-range sonar sensors n° 2 webcams to provide environmental feedback to the controller.	n.d.
Reshmi, et al., 2013 [31]	LH RH RLH RLF RELAX	Move left Move right Go forward Go backward Stop	RMS EEG machine	NO	3: C3,C4, Cz	256	PSD	SVM	INDOOR (Laboratory) Average duration: 2.30 min each run	50 CTR	n.d.	Wheelchair Prototype with components ATMEGA 328 microcontroller L293 motor driving circuit	NO	Matlab
Carra et al., 2013 [32]	RH F	Forward Turn right	EEG device (model n.d.)	NO	6: F3, P3, Fz, Pz, F4, P4	256	BPM	LDA	INDOOR (Office/Laboratory) Average duration: 5 min (training test) + 3 sessions (7 positions each)	1 CTR	Average hit rate: 65.7%	Motorized wheelchair (model n.d.)	NO	LabView 9.0 Matlab
Li et al., 2013 [33]	RH LH RLF	Turn right Turn left Go forward	g.tec amplifier (Guger Technologies, Austria)	NO	14: C5, C3, C1, Cz, C2, C4, C6, CP5, CP3, CP1, CPz, CP2, CP4, CP6	256	CSP	SVM	INDOOR (Office) Average duration: 4 s (trial) * 12 (n° of trials) * 4 (n° of sessions)	3 CTR	Average trial accuracy: 82.56%	Wheelchair system (model n.d.)	NO	Provided GUI
Choi et al., 2012 [34]	LH (imagine clenching the left hand) RH (imagine squeezing the right hand) RLF STOP: EMG	Turns left Turns right Moves forward	g.tec system: an EEG cap and a gUSBamp amplifier (Guger Technologies, Schiedlberg, Austria)	EMG	5: C3, C4, Cz, FC3, FC4	256	CSFSD	SVM	INDOOR (Office) Average duration: Bar-controlling experiment: 5 s (trial) * 30 (n° of trials) * 7 (n° of sets) Obstacle avoidance experiment: 24–28 s (trial) * 10 (n° of trials) * 7 (n° of sets)	3 CTR	Success rate: 90–95%	Rear-wheel drive type wheelchair: JW active model of Yamaha Motor Co	NO	Matlab
Carrino et al., 2012 [35]	RH LH	Turn right Turn left	Emotiv Epoc	NO	14 (+2 ref): AF3; F7; F3; FC5; T7; P7; O1; O2; P8; T8; FC6; F4; F8; AF4 (+CMS and DRL)	128	n.d.	LDA	INDOOR (Office) Average duration: n.d.	1 CTR	Classification rate: 67.5–91% on 2 gestures (left and right inputs)	Wheelchair prototype	NO	Developed application GERBIL. OpenVibe
Tsui, et al., 2011 [36]	RH LH IDLE STATE	Turn right Turn left	g. tec amplifier (Guger Technologies, Schiedlberg, Austria)	NO	10 (5 bipolar EEG channels): C3 (FC3 vs. CP3), C1 (FC1 vs. CP1), Cz (FCz vs. CPz), C2 (FC2 vs. CP2), and C4 (FC4 vs. CP4)	250	Logarithmic Band Power	LDA	INDOOR (University of Essex’s robotic arena) Average duration: −108.75 s for subject 1–114.75 s for subject 2.	2 CTR	n.d.	Electric-powered wheelchair (RoboChair) with components: An on-board DSP TMS320LF2407-based controller for motion control of 2 differentially-driven wheels; An on-board embedded PC connected to the DSP motion controller via a USB link A 24-volt battery providing power for the DSP controller, the PC and drive motors A local joystick controller connected to an A/D converter of the DSP-based controller	n° 6 ultrasonic range sensors for obstacle avoidance; n° 1 Hokuyo URG-04LX laser range finder to scan the environment.	n.d.

Associated acronyms: RH: Right Hand; LH: Left Hand; RF: Right Foot; LF: Left Foot; RLF: Right and Left Foot; F: Foot; EMG: Electromyography; ECG: Electrocardiography; US: Ultrasound; LDA: Linear Discriminant Analysis; OVR: one versus rest; PSD: power spectral density; KNN: K-nearest neighbor; ANN: Artificial Neural Network; CSFSD: common spatial frequency subspace decomposition; WT: Wavelet Transform; FFT: Fourier Transform; BPM: Band Power Method; GUI: Graphical User Interface; IDE: Integrated Development Environment; CTR: control subjects; VE: Virtual Environment; s: seconds; min: minute; ref: reference; n.d: not defined. * In this paper, author presented an intelligent wheelchair that combines an MI- (or, alternatively, P300-) based BCI and an automated navigation system. Only the MI- based BCI solution is taken into account for the scope of the review. ^§^ Additional information regarding performance evaluation and metrics used can be found in Section 6. MI-BCWs performance evaluation.

## Data Availability

Not applicable.
